# Efficacy and Safety of IncobotulinumtoxinA for Treatment of Sialorrhea: A Multicenter, Phase 3 Study in Japan

**DOI:** 10.1002/mdc3.70259

**Published:** 2025-08-07

**Authors:** Nobutaka Hattori, Yohei Mukai, Noriko Nishikawa, Kazuko Hasegawa, Masahiko Tomiyama, Yasuyoshi Kimura, Yoshio Tsuboi, Ryosuke Takahashi, Ryota Nakamura, Yuishin Izumi, Hirohisa Watanabe, Morinobu Seki, Kenji Sekiguchi, Shohei Tateishi, Yusaku Matsushita, Yusaku Nakamura

**Affiliations:** ^1^ Department of Neurology Juntendo University Faculty of Medicine Tokyo Japan; ^2^ Department of Neurology National Center of Neurology and Psychiatry Tokyo Japan; ^3^ Department of Neurology National Hospital Organization Sagamihara National Hospital Sagamihara Japan; ^4^ Department of Neurology Hirosaki University Graduate School of Medicine Hirosaki Japan; ^5^ Department of Neurology Osaka University Graduate School of Medicine Osaka Japan; ^6^ Department of Neurology Fukuoka University Fukuoka Japan; ^7^ Department of Neurology Kyoto University Graduate School of Medicine Kyoto Japan; ^8^ Department of Neurology Juntendo University Urayasu Hospital Urayasu Japan; ^9^ Department of Neurology Tokushima University Graduate School of Biomedical Sciences Tokushima Japan; ^10^ Department of Neurology Fujita Health University School of Medicine Toyoake Japan; ^11^ Department of Neurology Keio University School of Medicine Tokyo Japan; ^12^ Division of Neurology Kobe University Graduate School of Medicine Kobe Japan; ^13^ Clinical Development Control Department Teijin Pharma Limited Tokyo Japan; ^14^ Clinical Development Department Teijin Pharma Limited Tokyo Japan; ^15^ Department of Neurology Rinku General Medical Center Osaka Japan; ^16^ Present address: Yoshio Tsuboi: Tsutsumi Clinic Fukuoka Parkinson's Disease Center Fukuoka Japan; ^17^ Present address: Juntendo University Graduate School of Medicine Tokyo Japan; ^18^ Present address: Research Administration Center Kyoto University (KURA) Kyoto Japan

**Keywords:** botulinum toxins, Parkinson's disease, drooling, hypersalivation, salivary gland

## Abstract

**Background:**

Sialorrhea, caused by various neurological diseases, impairs patient health and quality of life. After the results of a randomized controlled trial, incobotulinumtoxinA was approved for the treatment of chronic sialorrhea in the United States and Europe; however, no pharmacotherapy has been approved in Japan.

**Objective:**

The aim was to evaluate the efficacy and safety of incobotulinumtoxinA treatment for chronic sialorrhea in a single‐arm phase 3 study in Japan.

**Methods:**

Patients with chronic sialorrhea caused by neurological diseases (Parkinson's disease, atypical parkinsonism, and stroke, group A) and broader diseases (eg, muscular dystrophy and amyotrophic lateral sclerosis, group B) were enrolled. IncobotulinumtoxinA 100 U was injected into the salivary glands once every 16 weeks for 48 weeks. A primary endpoint was assessed in group A, whereas secondary endpoints and safety were assessed in both groups.

**Results:**

From November 2021 to August 2023, 92 patients (58 and 34 in groups A and B, respectively) received incobotulinumtoxinA at 28 institutions. The primary endpoint, the least square mean (standard error) of change in unstimulated salivary flow rate from baseline to 4 weeks, was −0.08 (0.009, 95% confidence interval [CI]: −0.10, −0.06) g/min, achieving the prespecified efficacy criteria (upper limit of the 95% CI <−0.04). The secondary endpoints were consistent across efficacy measures, indicating that reduced salivary secretion and improved drooling symptoms persisted for 48 weeks. The most common adverse events were dry mouth and dysphagia.

**Conclusions:**

The first study in Japan confirmed the efficacy of incobotulinumtoxinA treatment for chronic sialorrhea with good patient tolerability and no new safety concerns.

## Graphical abstract

This study demonstrated significant treatment efficacy and safety of incobotulinumtoxinA injection in Japanese patients with chronic sialorrhea caused by Parkinson's disease and other neurological conditions. Suppression of salivary secretion and improvement in drooling symptoms were also observed throughout 48 weeks. Treatment was well tolerated, and no new safety concerns were observed.

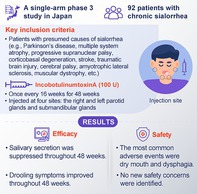

Sialorrhea is a chronic pathological condition in which hypersecretion or dysphagia drives saliva overflow from the oral cavity.^1^, ^2^, ^3^, ^4^, ^5^, ^6^, ^7^ Symptoms can range from wet lips to overflow at night/day, staining clothing and surrounding objects. Excessive saliva can cause skin sores around the lips, as well as dehydration and aspiration pneumonia. Neurological diseases such as Parkinson's disease (PD), atypical parkinsonism, stroke, traumatic brain injury, cerebral palsy, and amyotrophic lateral sclerosis (ALS) can lead to chronic sialorrhea due to neuromuscular dysfunction. Sialorrhea is commonly associated with PD, with prevalence rates ranging from 10% to 84%.^8^ The burden of Sialorrhea affects patients' health status and quality of life (QoL),^9^ as well as that of caregivers,^10^ ranging from difficulty eating and speaking with social and emotional consequences^11^ to increased risk of morbidity and mortality related to perioral skin breakdown and aspiration pneumonia.

Pharmacotherapy with botulinum toxins and anticholinergics is recommended for sialorrhea.^12^, ^13^ Cholinergic muscarinic receptor antagonists might cause adverse systemic reactions.^12^ Surgical and radiation therapies are invasive, whereas evidence for exercise or speech therapy is insufficient. Botulinum toxins act on peripheral cholinergic nerve endings, inhibiting acetylcholine release; when injected into the salivary glands, they can inhibit saliva secretion.^14^ The Sialorrhea in Adults Xeomin Investigation (SIAXI) study,^15^, ^16^ a phase 3 randomized, placebo‐controlled trial, conducted in Europe demonstrated that incobotulinumtoxinA 100 U is effective and well tolerated in adult patients with chronic sialorrhea. Subsequently, incobotulinumtoxinA was approved for chronic sialorrhea in the United States and in Europe in 2018 and 2019, respectively.^17^, ^18^ However, in Japan, no pharmacotherapy has been approved for sialorrhea. Furthermore, previous studies had limited representation of Asian populations, resulting in a lack of region‐specific evidence on the use of incobotulinumtoxinA for sialorrhea in Asians.^15^, ^16^ Additionally, the SIAXI study included only patients with PD, atypical parkinsonism, stroke, or traumatic brain injury and excluded individuals whose unstimulated salivary flow rate (uSFR) could not be measured.^15^, ^16^ Therefore, we conducted a multicenter clinical study in Asia to evaluate the efficacy and safety of incobotulinumtoxinA in a broader and more inclusive population, including patients with chronic sialorrhea who were not represented in the SIAXI trials.

## Patients and Methods

### Study Design

This multicenter, prospective, open‐label, single‐arm, 48‐week phase 3 study was conducted at 28 institutions in Japan from November 2021 to August 2023. Because the efficacy and safety of incobotulinumtoxinA are not influenced by endogenous or exogenous ethnic factors, the results of the SIAXI^15^, ^16^ can be extrapolated to other regions or other races; therefore, no placebo group was established in this study.

### Study Patients

Adult patients aged 20 to 80 years with chronic sialorrhea and diseases or conditions presumed to cause sialorrhea were enrolled. Patients fulfilling the following criteria continuously for ≥12 weeks were enrolled: sum score ≥6 points for the Drooling Severity and Frequency Scale (DSFS),^19^ score ≥2 points for each subscale of the DSFS, and score ≥3 points on the “drooling severity” of the modified version^16^ of the Radboud Oral Motor Inventory for Parkinson's Disease^20^ (mROMP).

In this clinical study, 2 groups of patients, groups A and B, were studied. Group A was established to evaluate the efficacy and safety of incobotulinumtoxinA in Japanese patients with chronic sialorrhea with the same causative diseases as in the SIAXI.^15^ Group B was established to enroll patients who did not meet the inclusion criteria for the SIAXI study and to evaluate secondary endpoints and safety. Group B comprised patients who could not undergo uSFR or other tests of group A and patients excluded from group A (muscular dystrophy, ALS, etc.). The full inclusion and exclusion criteria for groups A and B are described in the Supporting Information (Supplement S1). The main inclusion criteria for group A were as follows: diagnosis of idiopathic or familial PD, atypical parkinsonism (multiple system atrophy, progressive supranuclear palsy, or corticobasal degeneration), stroke, or traumatic brain injury; and ability to comply with the assessment schedule, including evaluation of the primary endpoint (uSFR). The main exclusion criteria for patients in groups A and B were scores ≥3 and ≥4 points on the mROMP swallowing symptom items A and C, respectively, or comorbid systemic neuromuscular junction disorders (eg, myasthenia gravis, Lambert–Eaton syndrome).

### Study Treatment

After consent was obtained, preenrollment, and baseline examinations, incobotulinumtoxinA (Merz Pharma GmbH & Co. KGaA, Frakfurt, Germany) was administered to patients thrice for 48 weeks, once every 16 ± 2 weeks. IncobotulinumtoxinA (100 U) was dissolved in 2.0 mL of saline and injected (guided by ultrasound or anatomical landmarks) at 4 sites: 30 U (0.6 mL) in the right and left parotid glands and 20 U (0.4 mL) in the right and left submandibular glands.

Patients in group A evaluated for the primary endpoint were not allowed to receive any of the following treatments: head and neck surgery and/or radiotherapy for sialorrhea, botulinum toxins, anticoagulants, or medications that can cause drooling (eg, clozapine) or reduce saliva production (eg, fesoterodine fumarate). Antiparkinsonian drugs (including anticholinergics for the treatment of parkinsonism) were allowed, as long as the dosage and administration were maintained.

### Efficacy Measure

The following measures were assessed to evaluate efficacy in group A: uSFR, DSFS, Global Impression of Change Scale (GICS), mROMP drooling and speech symptoms, and QoL using the EuroQol 5 dimensions 5 levels (EQ‐5D‐5L). In group B, only the DSFS and GICS were assessed to reduce the patient burden. In the measures of the GICS, mROMP, and EQ‐5D‐5L, if the patient was unable to record the answers, the caregiver assisted in recording them.

The uSFR (g/min) was measured via direct saliva collection using the swab method. Four oral swabs (2‐mL capacity; Salimetrics, Carlsbad, CA, USA) were placed in the patient's mouth for 5 min, and the differences in weights before and after placement were measured. The average of the results of 2 measurements taken every 30 min was calculated.

The DSFS was measured using 2 subscales: drooling severity on a 5‐point Likert scale from 1 (dry: never drools) to 5 (profuse: hands, trays, and objects wet), and drooling frequency on a 4‐point Likert scale from 1 (never) to 4 (constantly). The score (ranging from 2 to 9) was calculated as the sum of 2 subscales. The GICS^21^ score was measured on the following 7‐point Likert scale: −3 (very much worse), −2 (much worse), −1 (minimally worse), 0 (no change), +1 (minimally improved), +2 (much improved), and +3 (very much improved). Respondents provided their general impression of how the patient's condition had changed after treatment compared to the last administration of the drug. The mROMP drooling and speech symptoms^16^ were measured using a 24‐item questionnaire of a 5‐point Likert scale from 1 (normal) to 5 (worst). Patients recorded their answers regarding their condition over the past 7 days. QoL was measured using the EQ‐5D‐5L questionnaire.^22^ EQ‐5D‐5L comprises 2 parts: a descriptive system comprising 5 dimensions (mobility, self‐care, usual activities, pain/discomfort, and anxiety/depression) answered at 5 levels (from “no problems” to “extreme problems”) and a Visual Analog Scale (VAS) with self‐rating from 0 (the worst health you can imagine) to 100 (the best health you can imagine).

### Safety Measure

The safety of incobotulinumtoxinA was evaluated in patients in groups A and B during the treatment. For safety, the occurrence of adverse events (AEs), treatment‐related AEs (TRAEs), and serious AEs based on the Medical Dictionary of Regulatory Activities Japanese translation (MedDRA/J), version 26.0, was recorded by the investigators. Laboratory test results and vital signs were also monitored. Anti‐botulinum toxin A antibodies (requiring blood sampling) were assessed in patients in group A but not in group B to reduce patient burden.

### Statistical Analysis

The primary endpoint was the change in uSFR (g/min) from baseline to 4 weeks after the first administration of incobotulinumtoxinA in group A. For primary analysis, a mixed model for repeated measures analysis was performed, with changes in uSFR at 4 weeks as the objective variable, baseline uSFR as the explanatory variable, and the time point of examination as a fixed effect. The least square (LS) means and 95% confidence intervals (CIs) were calculated for each time point. The LS mean of the change in uSFR after 4 weeks in the placebo group in the SIAXI^15^ was −0.04; therefore, the threshold for the efficacy criterion was prespecified as −0.04 g/min. Treatment with incobotulinumtoxinA was determined to be effective if the upper limit of the 95% CI of the change in uSFR after 4 weeks was below that threshold.

The LS mean of the change in uSFR (g/min) after 4 weeks in the SIAXI^15^ was −0.13 and −0.04 in the incobotulinumtoxinA 100 U and placebo groups, respectively; as such, the expected value in this study was set at −0.13, the threshold at −0.04, and the standard deviation (SD) at 0.21. Assuming a 1‐sample *t*‐test at the 1‐sided significance level of 2.5% and a power of 80%, the required number of patients was 45. Considering dropout, the sample size for efficacy evaluation was set at 50 patients (group A). To evaluate the safety of incobotulinumtoxinA for 48 weeks in as many patients as possible, considering feasibility, the sample size for safety evaluation was set at 80 patients (groups A and B).

For sensitivity analysis of the primary endpoint, the means and 95% CIs based on a 1‐sample *t*‐test were calculated. Missing data were handled using baseline observation carried forward analysis and observed case analysis (analysis using the data obtained). For the primary endpoint, means and 95% CIs based on 1‐sample *t*‐tests were calculated for each subgroup according to 19 predefined characteristics. Descriptive analyses were performed for baseline patient characteristics, the secondary endpoints (uSFR, GICS, DSFS, mROMP, and EQ‐5D‐5L), and safety over the 48‐week study period. Statistical analyses were performed using SAS, version 9.4 (SAS Institute, Cary, NC, USA).

## Results

### Study Patients and Treatments

**FIG. 1:**
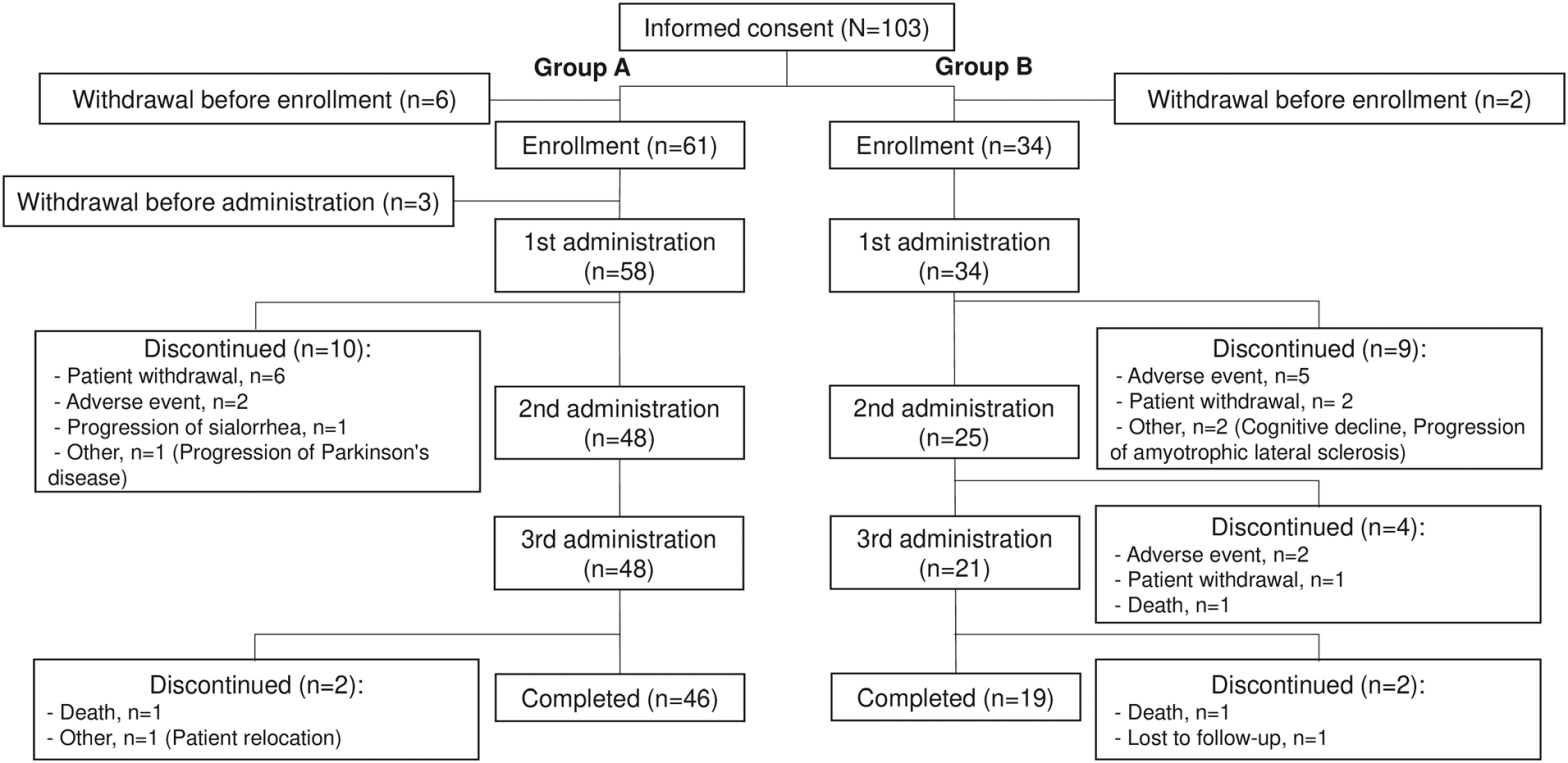
Patient flow diagram. Group A was established to evaluate the efficacy and safety of incobotulinumtoxinA in Japanese patients with chronic sialorrhea with the same causative diseases as in the SIAXI (Sialorrhea in Adults Xeomin Investigation).^15^ Group B was established to evaluate the secondary endpoints and safety. Group B comprised patients who could not undergo uSFR (unstimulated salivary flow rate) or other tests of group A and patients excluded from group A (muscular dystrophy, amyotrophic lateral sclerosis, etc.). The safety analysis set consisted of patients who received at least 1 administration of incobotulinumtoxinA (group A, N = 58; group B, N = 34). The full analysis set (for efficacy analysis) consisted of patients who were included in the safety analysis set, excluding 1 patient (group A) with no data on uSFR (group A, N = 57; group B, N = 34).

All 95 patients enrolled in the study were Japanese (Asian race), with 61 in group A and 34 in group B (Fig. 1). Of these patients, 92 received the first administration of incobotulinumtoxinA (group A: 58 and group B: 34 patients), 73 the second administration (48 and 25 patients), and 69 the third administration (48 and 21 patients). The injections were administered under ultrasound guidance in 86 patients (94.5%) and under anatomical landmark guidance in 5 patients (5.5%) at the first administration, and similarly at the second and third administrations. The most frequent reasons for discontinuation were patient request for withdrawal in group A and AEs in group B. The 92 patients who received at least 1 administration of incobotulinumtoxinA were included in the safety analysis, and 91 patients (57 and 34 patients) were included in the efficacy analysis (full analysis set), excluding 1 patient from group A because uSFR was not measured.

In the full analysis set, 69 (75.8%) patients were male, with a mean (SD) age of 67.5 (11.5) years (Table 1). The mean (SD) duration of sialorrhea was 28.3 (27.4) months in group A and 46.1 (79.9) months in group B. In group A, diseases causing sialorrhea were PD in 50 patients (87.7%), atypical parkinsonism in 4 patients (7.0%), and stroke in 3 patients (5.3%). One patient with a stroke also had symptomatic epilepsy and aphasia. None of the patients had traumatic brain injury. In group B, diseases causing sialorrhea were PD in 25 patients (73.5%); muscular dystrophy in 3 patients (8.8%); ALS in 2 patients (5.9%); and atypical parkinsonism, cerebral palsy, congenital myopathy, and multiple sclerosis in 1 patient each (2.9%).

**TABLE 1:** Baseline demographics and patient characteristics (full analysis set)

	Group A (N = 57)	Group B (N = 34)	Total (N = 91)
Male sex, n (%)	43 (75.4)	26 (76.5)	69 (75.8)
Age (yr), mean (SD)	67.6 (9.7)	67.5 (14.2)	67.5 (11.5)
Weight^a^ (kg), mean (SD)	58.5 (11.9)	–	–
Diseases causing sialorrhea^b^, n (%)			
Parkinson's disease	50 (87.7)	25 (73.5)	75 (82.4)
Atypical parkinsonism	4 (7.0)	1 (2.9)	5 (5.5)
Multiple system atrophy	1 (1.8)	0	1 (1.1)
Corticobasal degeneration	0	0	0
Progressive supranuclear palsy	3 (5.3)	1 (2.9)	4 (4.4)
Stroke, n (%)	3 (5.3)	0	3 (3.3)
Traumatic brain injury	0	0	0
Cerebral palsy	0	1 (2.9)	1 (1.1)
Amyotrophic lateral sclerosis	0	2 (5.9)	2 (2.2)
Muscular dystrophy	0	3 (8.8)	3 (3.3)
Other^c^	1 (1.8)	2 (5.9)	3 (3.3)
Duration of sialorrhea^d^ (mo), mean (SD)	n = 29	n = 16	n = 45
	28.3 (27.4)	46.1 (79.9)	34.6 (52.2)
Duration of diseases causing sialorrhea^e^ (mo), mean (SD)	n = 23	n = 14	n = 37
	101.2 (49.6)	198.4 (149.1)	138.0 (108.7)
Duration of Parkinson's disease (mo), mean (SD)	n = 19	n = 8	n = 27
	97.5 (46.5)	160.4 (80.3)	116.1 (64.0)
Dose of antiparkinsonian drugs^f^ (mg), mean (SD)	n = 50	n = 25	n = 75
	930.5 (462.5)	816.3 (336.5)	892.5 (425.8)
<600 mg, n (%)	11 (22.0)	8 (32.0)	19 (25.3)
≥600 mg, n (%)	39 (78.0)	17 (68.0)	56 (74.7)

*Notes*: Group A was established to evaluate the efficacy and safety of incobotulinumtoxinA in Japanese patients with chronic sialorrhea with the same causative diseases as in the SIAXI.^15^ Group B was established to evaluate the secondary endpoints and safety. Group B comprised patients who could not undergo uSFR or other tests of group A and patients excluded from group A (muscular dystrophy, amyotrophic lateral sclerosis, etc.).

Abbreviations: SD, standard deviation; SIAXI, Sialorrhea in Adults Xeomin Investigation; uSFR, unstimulated salivary flow rate.

^a^ Patients in group B were not weighed.

^b^ Multiple entries possible.

^c^ One patient with symptomatic epilepsy and aphasia in group A (this patient also had a stroke) and 1 patient each with congenital myopathy and multiple sclerosis in group B.

^d^ Duration of sialorrhea (months) = [before registration test (date) – onset of sialorrhea (date) + 1]/30.5.

^e^ Duration of diseases causing sialorrhea (months) = [before registration test (date) − onset of the disease (date) + 1]/30.5.

^f^ Levodopa equivalent daily dose.

Five of the 92 patients (5.4%) in the safety analysis set previously received botulinum toxin preparations for treatment other than sialorrhea. During the study period, 89 patients (96.7%) received concomitant medications, the most common of which were antiparkinsonian drugs (80 patients, 87.0%), systemic antibacterials (26 patients, 28.3%), and antiepileptic drugs (25 patients, 27.2%). A visual summary of the study design and key findings is included as graphical abstracts, presented in English language in the main article and in Japanese language in the Supplementary Material (Data S1).

### Efficacy

#### Unstimulated Salivary Flow Rate

**FIG. 2:**
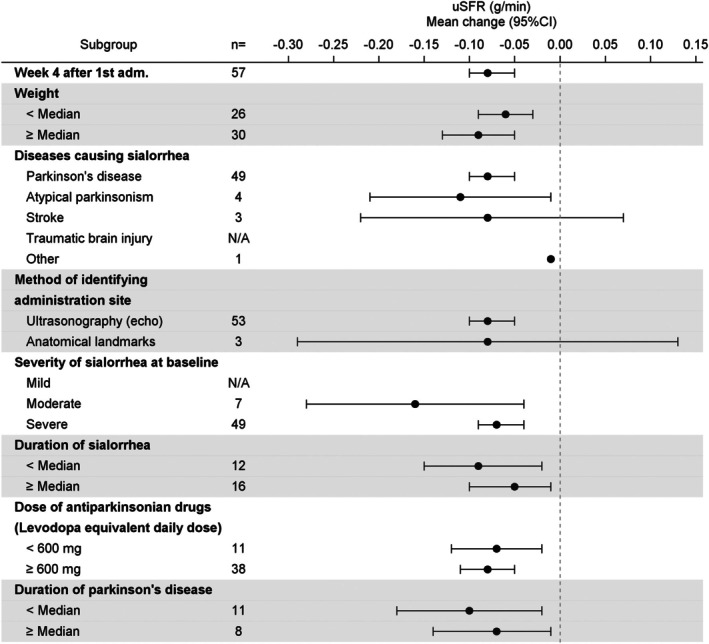
Subgroup analysis of the primary endpoint, changes in uSFR (unstimulated salivary flow rate, g/min) from baseline to 4 weeks after the first administration of incobotulinumtoxinA (group A). “Diseases causing sialorrhea” allowed multiple entries within a patient. Mild, moderate, and severe for “severity of sialorrhea at baseline” represent DSFS (Drooling Severity and Frequency Scale) sum score of 2 to 4, 5 to 6, and 7 to 9, respectively. Group A was established to evaluate the efficacy and safety of incobotulinumtoxinA in Japanese patients with chronic sialorrhea with the same causative diseases as in the SIAXI (Sialorrhea in Adults Xeomin Investigation).^15^ N/A indicates “no applicable patients.” CI, confidence interval.

**FIG. 3:**
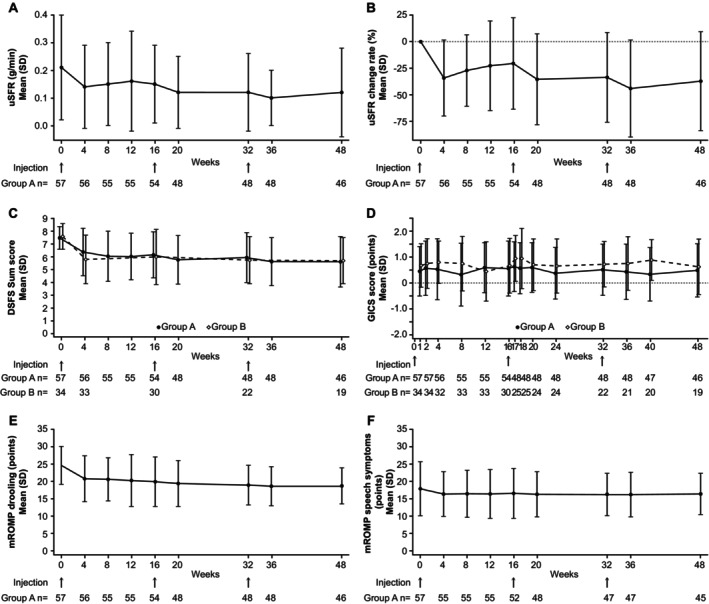
The secondary endpoints of efficacy during treatment with incobotulinumtoxinA. Arrows for the “injection” indicate the administration of incobotulinumtoxinA 100 U. (**A**) uSFR (unstimulated salivary flow rate, group A), (**B**) percentage change in uSFR (group A), (**C**) DSFS (Drooling Severity and Frequency Scale, groups A and B), (**D**) GICS (Global Impression of Change Scale, groups A and B), and (**E**) mROMP (modified version of the Radboud Oral Motor Inventory for Parkinson's Disease) drooling and (**F**) speech symptoms (group A). In the GICS and mROMP by patients, if the patient was unable to record answers, the caregiver assisted in recording them. Group A was established to evaluate the efficacy and safety of incobotulinumtoxinA in Japanese patients with chronic sialorrhea with the same causative diseases as in the SIAXI (Sialorrhea in Adults Xeomin Investigation).^15^ Group B was established to evaluate the secondary efficacy endpoints and safety. Group B comprised patients who could not undergo uSFR or other tests of group A and patients excluded from group A (muscular dystrophy, amyotrophic lateral sclerosis, etc.). SD, standard deviation.

In group A (n = 57), LS mean (standard error) of the change in uSFR from baseline to 4 weeks after the first administration, the primary endpoint, was −0.08 (0.009, 95% CI: −0.10, −0.06) g/min. The upper limit of the 95% CI (−0.06 g/min) was below the prespecified threshold for efficacy (−0.04 g/min); thus, the primary endpoint was achieved. The results of sensitivity analysis for the primary endpoint were similar to those of the primary analysis. The decrease in uSFR from baseline to 4 weeks was consistent considering patients' characteristics such as weight, etiology, severity, and duration of sialorrhea; injection guidance technique; and drug dose and duration of PD (Fig. 2). The mean (SD) uSFR (g/min) was 0.21 (0.19) at baseline. After the first administration, a decrease in the uSFR was observed at 4 weeks, persisting until 16 weeks. A similar persistent decrease in uSFR was observed after the second and third administrations, continuing for 48 weeks (the secondary endpoint, Fig. 3A,B).

#### Secondary Endpoints

##### Drooling Severity and Frequency Scale

Mean (SD) DSFS sum scores for groups A and B were 7.51 (0.89) and 7.62 (1.02) at baseline (study enrollment criterion was ≥6), respectively (Fig. 3C). A decrease in the DSFS sum scores was observed after 4 weeks and was sustained at 16 weeks. This decrease continued after the second and third administrations during the 48‐week study period.

##### Global Impression of Change Scale

Mean (SD) GICS scores for groups A and B after the first administration were 0.46 (0.96) and 0.68 (0.84) at 1 week and remained similar throughout the study period (Fig. 3D). The proportion of responders who reported a GICS score of 1 (minimally improved) or higher was 55.4% in group A and 75.0% in group B at 4 weeks and was generally above 50% during the study period. The results for patients and caregivers during the study period were similar in both groups (Fig. S1; Table S1).

##### Modified Version of the Radboud Oral Motor Inventory for Parkinson's Disease

In group A, the mean (SD) mROMP drooling and speech symptom scores were 24.68 (5.54) and 18.02 (7.73) at baseline, slightly decreasing to 20.80 (6.57) and 16.45 (6.39) at 4 weeks after the first administration, remaining similar during the study period (Fig. 3E,F).

##### EuroQol 5 Dimensions 5 Levels

Regarding anxiety/depression (one of the EQ‐5D‐5L single items), the proportion of patients with a score of 1 (eg, I am not anxious or depressed) increased after administration. No obvious changes were observed in other items (Table S2). The VAS scores improved slightly (Table S3).

### Safety

Of the 92 patients in the safety analysis set (58 and 34 patients in groups A and B, respectively), AEs and TRAEs were observed in 76 (82.6%) and 20 (21.7%) patients, respectively (Table 2). No increase in AEs was observed with continuing administration of incobotulinumtoxinA (Table S4). The most common TRAEs were dry mouth and dysphagia in 8 patients (8.7%) each (for both events, 4 patients each in groups A and B), as well as thirst in 3 patients (3.3%, 1 and 2 patients in groups A and B, respectively; Table S5).

**TABLE 2:** Number (%) of patients with adverse events during treatment with incobotulinumtoxinA over a 48‐week study period (safety analysis set)

	Overall: 48‐week study period		
	Group A (N = 58)	Group B (N = 34)	Total (N = 92)
Any adverse events	46 (79.3)	30 (88.2)	76 (82.6)
Any TRAEs	9 (15.5)	11 (32.4)	20 (21.7)
Serious			
Adverse events	11 (19.0)	10 (29.4)	21 (22.8)
TRAEs	2 (3.4)	1 (2.9)	3 (3.3)
Leading to discontinuation			
Adverse events	2 (3.4)	7 (20.6)	9 (9.8)
TRAEs	2 (3.4)	5 (14.7)	7 (7.6)
Leading to death			
Adverse events	1 (1.7)	2 (5.9)	3 (3.3)
TRAEs	0	0	0

*Notes*: Group A was established to evaluate the efficacy and safety of incobotulinumtoxinA in Japanese patients with chronic sialorrhea with the same causative diseases as in the SIAXI.^15^ Group B was established to evaluate the secondary endpoints and safety. Group B comprised patients who could not undergo uSFR or other tests of group A and patients excluded from group A (muscular dystrophy, amyotrophic lateral sclerosis, etc.). The Medical Dictionary for Regulatory Activities, version 26.0, was used for coding adverse events. TRAEs are adverse events determined to be related to the administration of incobotulinumtoxinA.

Abbreviations: TRAE, treatment‐related adverse event; SIAXI, Sialorrhea in Adults Xeomin Investigation; uSFR, unstimulated salivary flow rate.

Eleven AEs (2 and 9 events in groups A and B, respectively) occurred in 9 patients, leading to the discontinuation of incobotulinumtoxinA. Of these, 8 events in 7 patients were identified as TRAEs (dysphagia [2 events] in group A, dysphagia [4 events], and dry mouth and aspiration pneumonia [1 event each] in group B; Tables S6 and S7). Serious AEs were observed in 21 patients, of which events in 3 patients were identified as TRAEs (dysphagia in 2 patients in group A and aspiration pneumonia in 1 patient in group B; Table S8). Regarding AEs leading to death, sudden death (after the third administration) occurred in 1 patient in group A, and multiple organ dysfunction syndrome (after the first administration) and cardiac death (after the second administration) occurred in 1 patient each in group B. Causal relationships between these AEs and incobotulinumtoxinA treatment were ruled out. In group B, patients who experienced TRAEs during the study period classified by diseases causing sialorrhea were 5 of 25 patients with PD and all patients with muscular dystrophy (all 3 patients: dry mouth and dysphagia), ALS (both patients: eyelid ptosis, binocular eye movement disorder, and thirst), and congenital myopathy (1 patient: dysphagia).

No apparent changes in the laboratory test results or vital signs were observed. Anti‐botulinum toxin A antibodies were not detected in any patients at baseline or during the study period.

## Discussion

This study was conducted in accordance with the SIAXI,^15^ which showed a significant effect of incobotulinumtoxinA treatment on sialorrhea. IncobotulinumtoxinA treatment was as effective in Asian patients as the 100‐U group in the SIAXI.^15^ Although this was an open‐label study, the uSFR decreased beyond the threshold established based on the change in the placebo group in the SIAXI.^15^ To the best of our knowledge, this is the first clinical study in Asian patients to evaluate the efficacy and safety of incobotulinumtoxinA in the treatment of chronic sialorrhea caused by various neurological diseases. The primary endpoint of this study was achieved, demonstrating treatment efficacy of incobotulinumtoxinA. Several known side effects of incobotulinumtoxinA were observed, but no new side effects or complications were observed.

The efficacy results of this study, including the primary endpoint of change in the uSFR (representing saliva volume) at 4 weeks, confirmed the study hypothesis and were consistent with the SIAXI.^15^, ^16^ Patient characteristics were similar between this study and the SIAXI, except for the following: the drooling etiology was stroke in 5.3% (3/57 patients, group A) in this study compared to 18.9% in the SIAXI (14/74, incobotulinumtoxinA 100 U). The mean (SD) baseline uSFR (g/min) was 0.21 (0.19) in this study but was higher in the SIAXI at 0.40 (0.27). The LS mean of uSFR change at 4 weeks was −0.08 g/min in this study, and the upper limit of 95% CI (−0.06) satisfied the efficacy criteria (<−0.04). The SIAXI showed a slightly larger reduction, −0.13 g/min.^15^ However, additional analyses of these results with adjustments for baseline uSFR values showed no statistically significant differences between the 2 studies (data not shown). Our results (−0.08 g/min) revealed a larger reduction than that of the placebo group in the SIAXI (−0.04 g/min), supporting the saliva‐reducing effect of incobotulinumtoxinA. In addition, the subgroup analysis suggested that incobotulinumtoxinA may have a consistent effect regardless of patient characteristics (etiology, severity, and duration of sialorrhea, or duration and drug dose of PD). The secondary endpoints of this study, uSFR, mROMPs (assessed in group A), DSFS, and GICS (assessed in groups A and B), all exhibited a trend toward improvement from 4 weeks (1 week for GICS) after treatment initiation and were maintained throughout the 48‐week study period. These early‐onset and sustained effects of incobotulinumtoxinA are consistent with those reported for chronic sialorrhea^15^, ^16^, ^23^ and spasticity.^24^, ^25^

The favorable safety profile of incobotulinumtoxinA has generally been recognized,^26^ and the safety results of this study are consistent with these known profiles.^15^, ^16^, ^17^, ^18^, ^26^ The incidence of AEs was 46.7% after the first administration, 46.6% after the second administration, and 56.5% after the third administration in this study, which was similar to that reported in the SIAXI (total incobotulinumtoxinA [100 and 75 U]; 44.6%, 43.6%, and 35.6% in cycles 1, 2, and 3, respectively).^15^, ^16^ The most frequent TRAEs in this study were dry mouth and dysphagia, which paralleled those in the SIAXI.^15^, ^16^ The serious TRAEs of dysphagia (2 patients in group A) and aspiration pneumonia (1 patient in group B) were observed in this study. Patients in group B had a longer disease duration and more severe conditions that precluded the measurement of uSFR. Further, although the incidence of TRAEs was higher in group B than in group A, the types of events did not differ. In group B, treatment was discontinued due to TRAEs in 2 patients with PD (1 due to aspiration pneumonia and dysphagia, and 1 due to dry mouth), 2 patients with muscular dystrophy, and 1 patient with congenital myopathy (all these 3 due to dysphagia). A careful decision to start and/or continue incobotulinumtoxinA treatment considering each patient's risk of dysphagia is recommended. In the future, consideration of the site of administration and the total dosage may be required for such patients.

Sialorrhea remains poorly recognized in Asia, and there are currently no guidelines to define the diagnostic criteria for sialorrhea or methods for measuring salivary secretion. In addition, rating scales for drooling have not yet been established.^27^, ^28^ Following previous studies,^15^, ^16^ this is the first study in Asia to measure salivary secretion using swabs and assess several rating scales during sialorrhea treatment. Furthermore, group B in this study comprised patients not included in the SIAXI (eg, ALS),^15^ for whom previous reports of incobotulinumtoxinA treatment are scarce.^29^, ^30^ This study is also the first clinical trial of botulinum toxin in Asia for sialorrhea, including patients with ALS. Although the salivary secretion (uSFR) was not measured in group B, subjective measures such as the DSFS and GICS suggested an improvement in drooling after incobotulinumtoxinA administration. In this study, GICS were obtained not only from patients but also from caregivers. The responses were similar, indicating that the caregivers perceived that the patient's condition had improved after the initiation of treatment. Anxiety/depression items showed a tendency to improve, and the VAS also showed a slight improvement. The QoL of patients and caregivers during the sialorrhea treatment is an issue for future research.

The limitations of this study include its single‐arm design. Although the sample size required for the primary analysis of efficacy was achieved, the number of patients was limited based on the characteristics analyzed in the subgroups. In addition, 80% of the patients had PD as the causative disease, and the number of patients with diseases other than PD was small. This may be because the prevalence of the causative disease was higher than that of other diseases, whereas PD was easily applicable when patients with no (mild) dysphagia but with drooling were selected according to the inclusion and exclusion criteria. Future research should accumulate evidence for incobotulinumtoxinA treatment in patients with chronic sialorrhea from diverse backgrounds through larger studies.

In conclusion, this study demonstrated that incobotulinumtoxinA was effective and well tolerated in Asian patients with chronic sialorrhea, particularly in those with underlying conditions in the SIAXI trial, most notably PD.

## Author Roles

(1) Research project: A. Conception, B. Organization, C. Execution; (2) Statistical analysis: A. Design, B. Execution, C. Review and critique; (3) Manuscript preparation: A. Writing of the first draft, B. Review and critique.

N.H.: 1A, 1B, 1C, 3A, 3B

Y.Mu.: 1B, 1C, 3B

N.N.: 1B, 1C, 3B

K.H.: 1B, 1C, 3B

M.T.: 1B, 1C, 3B

Y.K.: 1B, 1C, 3B

Y.T.: 1B, 1C, 3B

R.T.: 1B, 1C, 3B

R.N.: 1B, 1C, 3B

Y.I.: 1B, 1C, 3B

H.W.: 1B, 1C, 3B

M.S.: 1B, 1C, 3B

K.S.: 1B, 1C, 3B

S.T.: 1A, 1B, 1C, 2A, 2B, 2C, 3A, 3B

Y.Ma.: 1A, 1B, 1C, 2A, 2C, 3A, 3B

Y.N.: 1A, 1B, 1C, 3A, 3B

## Disclosures

**Ethical Compliance Statement:** This study was approved by the institutional review board at each medical institution, conducted in accordance with the Declaration of Helsinki and Good Clinical Practice guidelines, and registered with the Japan Registry of Clinical Trials (jRCT2051210082). All patients or their representatives provided written informed consent to participate. We confirm that we have read the journal's position on issues involved in ethical publication and affirm that this work is consistent with those guidelines.

**Funding Sources and Conflicts of Interest:** This study was funded by Teijin Pharma Limited. Teijin Pharma was involved in this study and publication of this work. N.H. and N.N.'s institution had collaborative research programs with Teijin Pharma Limited. S.T. and Y.Ma. are employees of Teijin Pharma Limited. Y.N. received consultancy fees and honoraria from Teijin Pharma Limited. All other authors declare no competing interests relevant to this work.

**Financial Disclosures for the Previous 12 Months:** N.H. holds stocks of Parkinson Laboratories Co., Ltd. and received consultancy fees from Teijin Pharma Limited and Parkinson Laboratories Co., Ltd.; advisory board fees from Sumitomo Pharma Co., Ltd. and AbbVie GK; and grants from the Michael J. Fox Foundation. N.H.'s institution had collaborative research programs/endowed courses with Teijin Pharma Limited, Boston Scientific Corporation, Medtronic Japan Co., Ltd., Abbott Japan LLC, Apex Inc, Sumitomo Pharma Co., Ltd., Eisai Co., Ltd., Nihon Medi‐Physics Co., Ltd., Sunwels Co., Ltd., Ohara Pharmaceutical Co., Ltd., Parkinson Laboratories Co., Ltd., Ono Pharmaceutical Co., Ltd., Kyowa Kirin Co., Ltd., Mitsubishi Tanabe Pharma Corporation, Otsuka Pharmaceutical Co., Ltd., and Zebra Holdings Co., Ltd. Y.Mu. received honoraria from AbbVie GK, Teijin Pharma Limited, Mitsubishi Tanabe Pharma Corporation, Janssen Pharmaceutical K.K., Kyowa Kirin Co., Ltd., Sumitomo Pharma Co., Ltd., and Takeda Pharmaceutical Co., Ltd. N.N. received honoraria from Eisai Co., Ltd., Takeda Pharmaceutical Co., Ltd., AbbVie Inc., and Ono Pharmaceutical Co., Ltd., and grants from the Japan Society for the Promotion of Science under KAKENHI Grant Number JP24K09947 and the Japan Agency for Medical Research and Development under grant number JP24dk0207072h0001. N.N.'s institution had collaborative research programs with Teijin Pharma Limited, Eisai Co., Ltd., Nihon Medi‐Physics Co., Ltd., and Ono Pharmaceutical Co., Ltd. Y.K. received honoraria from Takeda Pharmaceutical Co., Ltd., Ono Pharmaceutical Co., Ltd., Eisai Co., Ltd., and AbbVie GK, and research funding from the Japan Society for the Promotion of Science under KAKENHI grant numbers JP21K15681 and JP24K10619 and the Japan Agency for Medical Research and Development under grant number JP22lk0201162. Y.T. received honoraria from Kyowa Kirin Co., Ltd., Takeda Pharmaceutical Co., Ltd., Sumitomo Dainippon Pharma Co., Ltd., and Eisai Co., Ltd., and grants from Sunwels Co., Ltd. R.T. received advisory board fees from Eisai Co., Ltd., Sumitomo Pharma Co., Ltd., GW Pharmaceuticals plc, Nippon Boehringer Ingelheim Co., Ltd., and Novartis Pharma K.K.; honoraria from Takeda Pharmaceutical Co., Ltd., Daiichi Sankyo Company, Limited, Kyowa Kirin Co., Ltd., Sumitomo Pharma Co., Ltd., AbbVie GK, Eisai Co., Ltd., Ono Pharmaceutical Co., Ltd., Nippon Boehringer Ingelheim Co., Ltd., CSL Behring K.K., Splink, Inc., Sunwels Co., Ltd., Chugai Pharmaceutical Co., Ltd., Planmake Co., argenx Japan K.K., Mitsubishi Tanabe Pharma Corporation, Otsuka Pharmaceutical Co., Ltd., EA Pharma Co., Ltd., FP Pharmaceutical Corporation, Alexion Pharmaceuticals, Inc., Novartis Pharma K.K., and Alnylam Japan K.K.; and grants from Nippon Boehringer Ingelheim Co., Ltd., Sumitomo Pharma Co., Ltd., and Otsuka Pharmaceutical Co., Ltd. H.W. received lecture fees from Kyowa Kirin Co., Ltd., Takeda Pharmaceutical Co., Ltd., Ono Pharmaceutical Co., Ltd., Sumitomo Pharma Co., Ltd., and Eisai Co., Ltd. M.S. received honoraria from AbbVie GK, Eisai Co., Ltd., Ono Pharmaceutical Co., Ltd., Kyowa Kirin Co., Ltd., Sumitomo Pharma Co., Ltd., and Takeda Pharmaceutical Co., Ltd., and grants from the Japan Society for the Promotion of Science (KAKENHI [C]) and the First Parkinson's Disease Quality of Life Foundation. S.T. and Y.Ma. are employees of Teijin Pharma Limited. Y.N. received consultancy fees and honoraria from Teijin Pharma Limited. All other authors declare no competing interests.

## Supporting information


**Supplement S1.** Inclusion and exclusion criteria of the study.
**Supplement S2.** Contributors of the study.
**Figure S1.** GICS scores by patients and caregivers (full analysis set, groups A and B). GICS, Global Impression of Change Scale; SD, standard deviation.
**Table S1.** GICS scores by patients and caregivers (full analysis set, groups A and B). GICS, Global Impression of Change Scale; SD, standard deviation.
**Table S2.** EQ‐5D‐5L (single items, full analysis set, group A). For mobility, 1: I have no problems in walking about, 2: I have slight problems in walking about, 3: I have moderate problems in walking about, 4: I have severe problems in walking about, 5: I am unable to walk about. For self‐care, 1: I have no problems washing or dressing myself, 2: I have slight problems washing or dressing myself, 3: I have moderate problems washing or dressing myself, 4: I have severe problems washing or dressing myself, 5: I am unable to wash or dress myself. For usual activities, 1: I have no problems doing my usual activities, 2: I have slight problems doing my usual activities, 3: I have moderate problems doing my usual activities, 4: I have severe problems doing my usual activities, 5: I am unable to do my usual activities. For pain/discomfort, 1: I have no pain or discomfort, 2: I have slight pain or discomfort, 3: I have moderate pain or discomfort, 4: I have severe pain or discomfort, 5: I have extreme pain or discomfort. For anxiety/depression, 1: I am not anxious or depressed, 2: I am slightly anxious or depressed, 3: I am moderately anxious or depressed, 4: I am severely anxious or depressed, 5: I am extremely anxious or depressed. adm., administration; EQ‐5D‐5L, EuroQol 5 dimensions 5 levels.
**Table S3.** EQ‐5D‐5L (Visual Analog Scale, full analysis set, group A). adm., administration; EQ‐5D‐5L, EuroQol, 5 dimensions 5 levels; SD, standard deviation; VAS, Visual Analog Scale.
**Table S4.** Number (%) of patients with adverse events during treatment with incobotulinumtoxinA after each stage of administration (safety analysis set). MedDRA, version 26.0, was used for coding adverse events. Treatment‐related adverse events are adverse events determined to be related to the administration of incobotulinumtoxinA. MedDRA, Medical Dictionary for Regulatory Activities.
**Table S5.** Treatment‐related adverse events in System Organ Class and Preferred Term of 2% or more (safety analysis set). Patients with 1 or more adverse events within a level of MedDRA term are counted only once in that level. Percentages are based on the number of patients in the safety analysis set for each group. MedDRA, version 26.0, was used for coding adverse events. MedDRA, Medical Dictionary for Regulatory Activities.
**Table S6.** Adverse events leading to discontinuation of incobotulinumtoxinA (safety analysis set). Patients with 1 or more adverse events within a level of MedDRA term are counted only once in that level. Percentages are based on the number of patients in the safety analysis set for each group. MedDRA, version 26.0, was used for coding adverse events. MedDRA, Medical Dictionary for Regulatory Activities.
**Table S7.** Treatment‐related adverse events leading to discontinuation of incobotulinumtoxinA (safety analysis set). Patients with 1 or more adverse events within a level of MedDRA term are counted only once in that level. Percentages are based on the number of patients in the safety analysis set for each group. MedDRA, version 26.0, was used for coding adverse events. MedDRA, Medical Dictionary for Regulatory Activities.
**Table S8.** Serious treatment‐related adverse events (safety analysis set). Patients with 1 or more adverse events within a level of MedDRA term are counted only once in that level. Percentages are based on the number of patients in the safety analysis set for each group. MedDRA, version 26.0, was used for coding adverse events. MedDRA, Medical Dictionary for Regulatory Activities.

**Data S1:** Graphical abstract (Japanese)

## Data Availability

Research data are not shared due to contract with Teijin Pharma Limited.
