# Supplementary material for: Efficacy and Safety of IncobotulinumtoxinA for Treatment of Sialorrhea: A Multicenter, Phase 3 Study in Japan
Source: Mov Disord Clin Pract. 2025 Aug 7;13(1):120–30. doi: 10.1002/mdc3.70259 (PMC12839494; doi:10.1002/mdc3.70259)
Supplement: Supplementary file 1 — Supplement S1. Inclusion and exclusion criteria of the study. Supplement S2. Contributors of the study. Figure S1. GICS scores by patients and caregivers (full analysis set, groups A and B). GICS, Global Impression of Change Scale; SD, standard deviation. Table S1. GICS scores by patients and caregivers (full analysis set, groups A and B). GICS, Global Impression of Change Scale; SD, standard deviation. Table S2. EQ‐5D‐5L (single items, full analysis set, group A). For mobility, 1: I have no problems in walking about, 2: I have slight problems in walking about, 3: I have moderate problems in walking about, 4: I have severe problems in walking about, 5: I am unable to walk about. For self‐care, 1: I have no problems washing or dressing myself, 2: I have slight problems washing or dressing myself, 3: I have moderate problems washing or dressing myself, 4: I have severe problems washing or dressing myself, 5: I am unable to wash or dress myself. For usual activities, 1: I have no problems doing my usual activities, 2: I have slight problems doing my usual activities, 3: I have moderate problems doing my usual activities, 4: I have severe problems doing my usual activities, 5: I am unable to do my usual activities. For pain/discomfort, 1: I have no pain or discomfort, 2: I have slight pain or discomfort, 3: I have moderate pain or discomfort, 4: I have severe pain or discomfort, 5: I have extreme pain or discomfort. For anxiety/depression, 1: I am not anxious or depressed, 2: I am slightly anxious or depressed, 3: I am moderately anxious or depressed, 4: I am severely anxious or depressed, 5: I am extremely anxious or depressed. adm., administration; EQ‐5D‐5L, EuroQol 5 dimensions 5 levels. Table S3. EQ‐5D‐5L (Visual Analog Scale, full analysis set, group A). adm., administration; EQ‐5D‐5L, EuroQol, 5 dimensions 5 levels; SD, standard deviation; VAS, Visual Analog Scale. Table S4. Number (%) of patients with adverse events during treatment with incobotulinumtoxinA after [file MDC3-13-120-s002.docx]

Supporting information

Table of contents

[1. **Supplement S1:** Inclusion and exclusion criteria of the study 2](#_Toc204786269)

[1.1. Inclusion criteria of the patients of the study 2](#_Toc204786270)

[**Inclusion criteria applied to groups A and B** 2](#_Toc204786271)

[**Inclusion criteria applied to group A only** 2](#_Toc204786272)

[**Inclusion criteria applied to group B only** 2](#_Toc204786273)

[1.2. Exclusion criteria of the patients of the study 3](#_Toc204786274)

[**Exclusion criteria applied to groups A and B** 3](#_Toc204786275)

[**Exclusion criteria applied to group A only** 4](#_Toc204786276)

[**Exclusion criteria applied to group B only** 4](#_Toc204786277)

[2. **Supplement S2**: Contributors of the study 5](#_Toc204786278)

[2.1. The study sites and investigators 5](#_Toc204786279)

[2.2. Uniformity of the assessments 5](#_Toc204786280)

[3. Tables and Figures 6](#_Toc204786281)

[**Fig. S1.** GICS scores by patients and caregivers (full analysis set, groups A and B) 6](#_Toc204786282)

[**Table S1.** GICS scores by patients and caregivers (full analysis set, groups A and B) 7](#_Toc204786283)

[**Table S2.** EQ-5D-5L (single items, full analysis set, group A) 8](#_Toc204786284)

[**Table S3.** EQ-5D-5L (Visual Analog Scale, full analysis set, group A) 10](#_Toc204786285)

[**Table S4.** Number (%) of patients with adverse events during treatment with incobotulinumtoxinA after each stage of administration (safety analysis set) 11](#_Toc204786286)

[**Table S5.** Treatment-related adverse events in System Organ Class and Preferred Term of 2% or more (safety analysis set) 12](#_Toc204786287)

[**Table S6.** Adverse events leading to discontinuation of incobotulinumtoxinA (safety analysis set) 13](#_Toc204786288)

[**Table S7.** Treatment-related adverse events leading to discontinuation of incobotulinumtoxinA (safety analysis set) 14](#_Toc204786289)

[**Table S8.** Serious treatment-related adverse events (safety analysis set) 15](#_Toc204786290)

# **Supplement S1:** Inclusion and exclusion criteria of the study

# Inclusion criteria of the patients of the study

### **Inclusion criteria applied to groups A and B**

The investigator shall select patients who meet all of the following criteria.

（1）Patients for whom written informed consent has been obtained from the patient or the legal representative before participating in the clinical trial.

（2）Men and women aged 20 to 80 years at the time of informed consent

（3）Patients who meet all of the following 1) to 3) at the preregistration examination

1）Drooling Severity and Frequency Scale (DSFS) sum score of 6 or more

2）DSFS scores of 2 or more for each item (severity and frequency)

3）modified version of the Radboud Oral Motor Inventory for Parkinson’s Disease (mROMP) Drooling A) (severity of sialorrhea) score of 3 or more

（4）Patients continuously meeting the inclusion criteria (3) from 12 weeks before the preregistration examination date. (It is allowed that the investigator confirms that sialorrhea symptoms of severity/frequency that meet the inclusion criteria (3) have continued for at least 12 weeks by interviewing the patients, etc., and leaves the results of the interview in the source documents.)

（5）Patients with presumed causes of sialorrhea (eg, Parkinson’s disease, atypical parkinsonism, stroke, traumatic brain injury, cerebral palsy, amyotrophic lateral sclerosis [ALS], muscular dystrophy, etc.)

Select persons who meet the following criteria for group A or group B.

### **Inclusion criteria applied to group A only**

Patients who meet 1) and 2) below

1）Patients who have been diagnosed with any of the following diseases i) to iv) and who have been diagnosed for at least 24 weeks since the onset of the disease by the date of the preregistration examination.

i）Idiopathic or familial Parkinson’s disease according to UK Parkinson’s Disease Brain Bank (UKPDSBB) diagnostic criteria^1^

ii）Clinically diagnosed atypical parkinsonism a) to c)

a) Multiple system atrophy

b) Progressive supranuclear palsy

c) Corticobasal degeneration

iii）Stroke

iv）Traumatic brain injury

2）Patients who are able to comply with the observation and examination schedules specified in group A, including unstimulated salivary flow rate (uSFR) measures

### **Inclusion criteria applied to group B only**

Patients not applicable to group A. However, after the sponsor has completed enrollment in group A, patients who fall into group A can also be enrolled in group B.

# Exclusion criteria of the patients of the study

The investigator shall check the exclusion criteria according to the selected group according to the inclusion criteria (5) and exclude any conflicting patients from the study.

### **Exclusion criteria applied to groups A and B**

（1）Patients with a score of 3 or more on mROMP Swallowing symptoms A) or a score of 4 or more on the C) regarding swallowing function at the preregistration examination. However, patients who use a tube feeding for nutritional support do not fall under mROMP Swallowing symptoms C) 5 “I had to use a feeding tube.”

（2）Patients who have experienced aspiration pneumonitis twice or more, patients who had the experience once and are judged by the investigator to be at increased risk of the recurrence, or patients who have not had gastrostomy after tracheostomy positive pressure ventilation (TPPV) induction.

（3）Patients with generalized neuromuscular junction disorders (such as myasthenia gravis and Lambert-Eaton syndrome)

（4）Patients with a history (or complication) of infection or tumor in the salivary glands or at the intended injection site. However, infections in childhood such as parotitis are permissible.

（5）Patients who are judged by the investigator to be at risk of preventing a safe participation in the clinical trial due to extremely poor dental oral health status

（6）Patients with concomitant malignant tumors or patients with a history of malignancy within 2 years of the date of preregistration examination.

（7）Patients with hypersensitivity to botulinum toxin (any toxin type) or drug additives (human serum albumin, purified white sugar)

（8）Patients with alcoholism or substance abuse or a history of alcoholism

（9）Women who are pregnant or possibly pregnant or breastfeeding

（10）Among women of childbearing potential, those who are unable to achieve effective contraception (eg, intrauterine device [IUD], intrauterine contraceptive system [IUS], oral contraceptive pill (low-dose pill), contraceptive surgery, double-barrier method [eg, condom with spermicide or condom combined with pessary]) under the guidance of the investigator during the period from the date of informed consent to the last observation.

（11）Patients who changed the dosage and administration of antiparkinsonian drugs* after 4 weeks prior to the preregistration examination date. However, administration of an L-dopa preparation to maintain constant motor symptoms is acceptable.

（12）Patients who have undergone surgical procedures within 4 weeks* prior to the date of the preregistration examination. However, minor procedures other than the head and neck are acceptable.

（13）Patients treated with botulinum toxin products within 14 weeks* prior to the preregistration examination date and (For patients who were treated with it for treatment of sialorrhea; within 1 year prior to the preregistration examination date).

（14）Patients who have received other investigational or unapproved drugs after 14 weeks* prior to the date of the preregistration examination

（15）Patients who have undergone functional neurosurgery (deep brain stimulation, stereotactic disruption) or focused ultrasound treatment within 24 weeks* prior to the date of the preregistration examination

（16）Patients who have previously undergone head and neck surgery or radiotherapy (eg, salivary gland surgery or salivary gland radiotherapy) for the treatment of sialorrhea

（17）Patients who are receiving muscle relaxants, drugs with muscle relaxant effect and anticoagulants. However, aspirin and antiplatelet agents are acceptable.

（18）Patients requiring use (implementation) of prohibited concomitant medication or change in dosage and administration of restricted concomitant medication after the test date before enrollment

### **Exclusion criteria applied to group A only**

（19）Patients who received drugs known to induce salivation (eg, clozapine) within 4 weeks* prior to the date of preregistration examination.

（20）Patients who received medications known to suppress salivation (eg, fesoterodine fumarate) within 4 weeks* prior to the date of preregistration examination. However, administration of anticholinergic drugs for the treatment of parkinsonism is acceptable in the case that the dosage and administration are constant.

（21）Patients who are unable to stay still for 5 minutes with the saliva collection swabs in their mouth

（22）Patients who are unable to open their mouth spontaneously

（23）Patients with Alzheimer’s disease or other dementias who are judged by the investigator to have an impact on the evaluation of the study

（24）Other than the above, patients who are judged by the investigator to be ineligible for the study (group A)

### **Exclusion criteria applied to group B only**

（25）Those who changed the dosage and administration of drugs known to induce salivation (such as clozapine) within 4 weeks* prior to the date of preregistration examination.

（26）Patients who changed the dosage and administration of drugs known to suppress salivation (eg, fesoterodine fumarate) within 4 weeks* prior to the date of examination prior to enrollment

（27）[Exclusion criteria only applicable to patients with ALS]

Patients who have a progressive decline in respiratory function and may receive noninvasive positive pressure ventilation (NPPV) or TPPV in 1 year. However, those who have already undergone TPPV are not eligible for this exclusion criterion.

（28）[Exclusion criteria only applicable to patients with ALS]

Patients whose percent forced vital capacity (%FVC) is <80% at the preregistration examination. However, those who have already undergone TPPV are not eligible for this exclusion criterion.

（29）Other than the above, patients who are judged by the investigator to be ineligible for the study (group B)

*It is defined as the day after the same day of the week prior to the specified period on the preregistration examination date.

Reference

1. parkinsons.org [Internet]. London, United Kingdom: Parkinson's UK. <https://www.parkinsons.org.uk>

# **Supplement S2**: Contributors of the study

# The study sites and investigators

National Center of Neurology and Psychiatry: Yohei Mukai, Madoka Mori, Toshiyuki Yamamoto, Hotake Takizawa

Juntendo University Hospital: Nobutaka Hattori, Noriko Nishikawa, Hikaru Kamo, Daiki Kamiyama

National Hospital Organization Sagamihara National Hospital: Kazuko Hasegawa, Aya Kawanami

Hirosaki University Hospital: Masahiko Tomiyama, Chieko Murakami, Haruo Nishijima, Tomoya Kon, Iku Kinoshita

Osaka University Hospital: Yasuyoshi Kimura, Hideki Mochizuki, Keita Kakuda

Fukuoka University Hospital: Yoshio Tsuboi

Kyoto University Hospital: Ryosuke Takahashi, Atsushi Shima, Etsuro Nakanishi

Juntendo University Urayasu Hospital: Ryota Nakamura, Naohide Kurita

Tokushima University Hospital: Yuishin Izumi, Kohei Muto

Fujita Health University Hospital: Hirohisa Watanabe, Yasuaki Mizutani, Kenichiro Murate, Atsuhiro Higashi

Keio University Hospital: Morinobu Seki, Toshiki Tezuka, Shohei Okusa

Kobe University Hospital: Kenji Sekiguchi, Takehiro Ueda, Kento Matoba

Sanyudo Rehabilitation Center: Masayuki Hosaka

National Hospital Organization Sendai Nishitaga Hospital: Atsushi Takeda, Kenichi Nagamatsu

Tsuchiura Kyodo General Hospital: Akira Machida

Tokyo Metropolitan Neurological Hospital: Hideki Kimura

National Hospital Organization Higashinagoya National Hospital: Ikuko Aiba, Yuki Yokokawa, Rina Hashimoto

Aichi Medical University Hospital: Hidemoto Saiki

National Hospital Organization Kumamoto Saishun Medical Center: Ryoichi Kurisaki, Yasushi Maeda, Yoshimune Yamasaki

Iwate Medical University Uchimaru Medical Center: Tetsuya Maeda

International University of Health and Welfare Narita Hospital: Eiichi Suehiro

Nara Medical University Hospital: Kazuma Sugie, Naohiko Iguchi

Kyushu Central Hospital: Hitonori Takaba

Kumamoto Takumadai Rehabilitation Hospital: Kenshi Obaru

Medical Corporation Sansyukai Okatsu Hospital: Kimiyoshi Arimura

National Hospital Organization Hokkaido Medical Center: Yuka Isikawa

Toho University Omori Medical Center: Takehisa Hirayama

# Uniformity of the assessments

1. Kindai University Hospital: Chiharu Isono

# Tables and Figures

## **Fig. S1.** GICS scores by patients and caregivers (full analysis set, groups A and B)


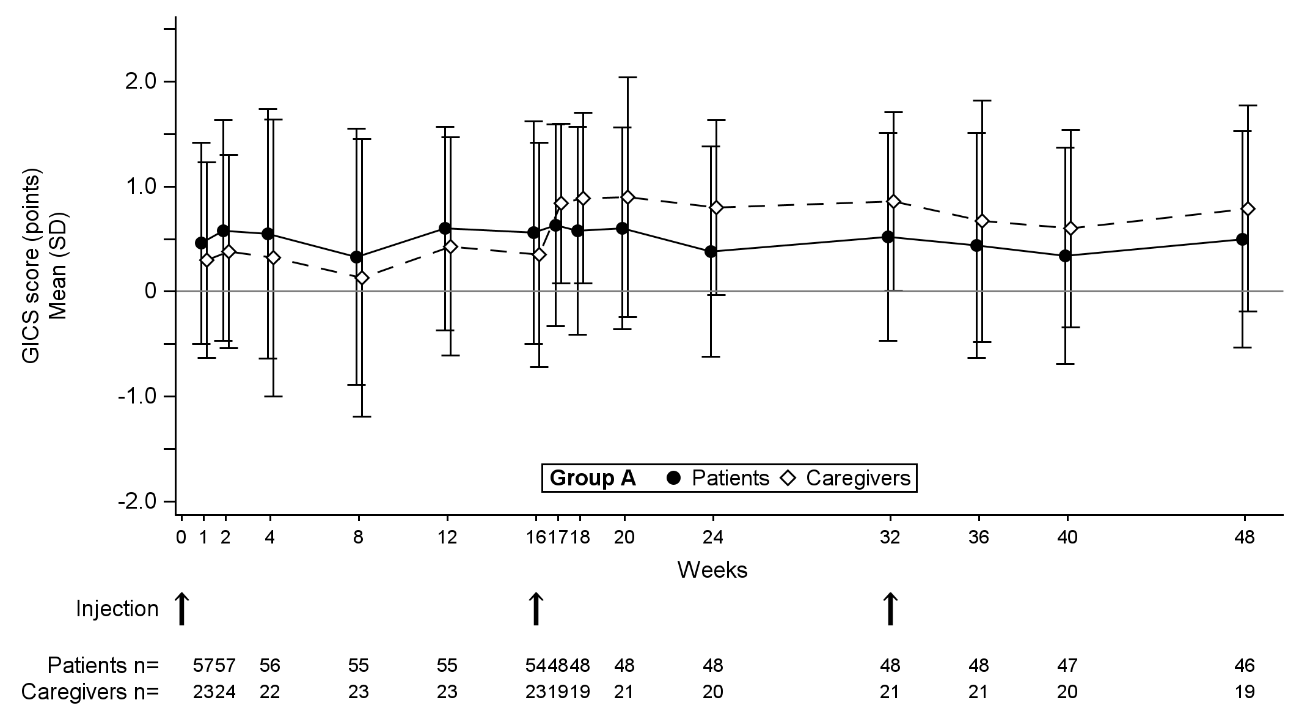


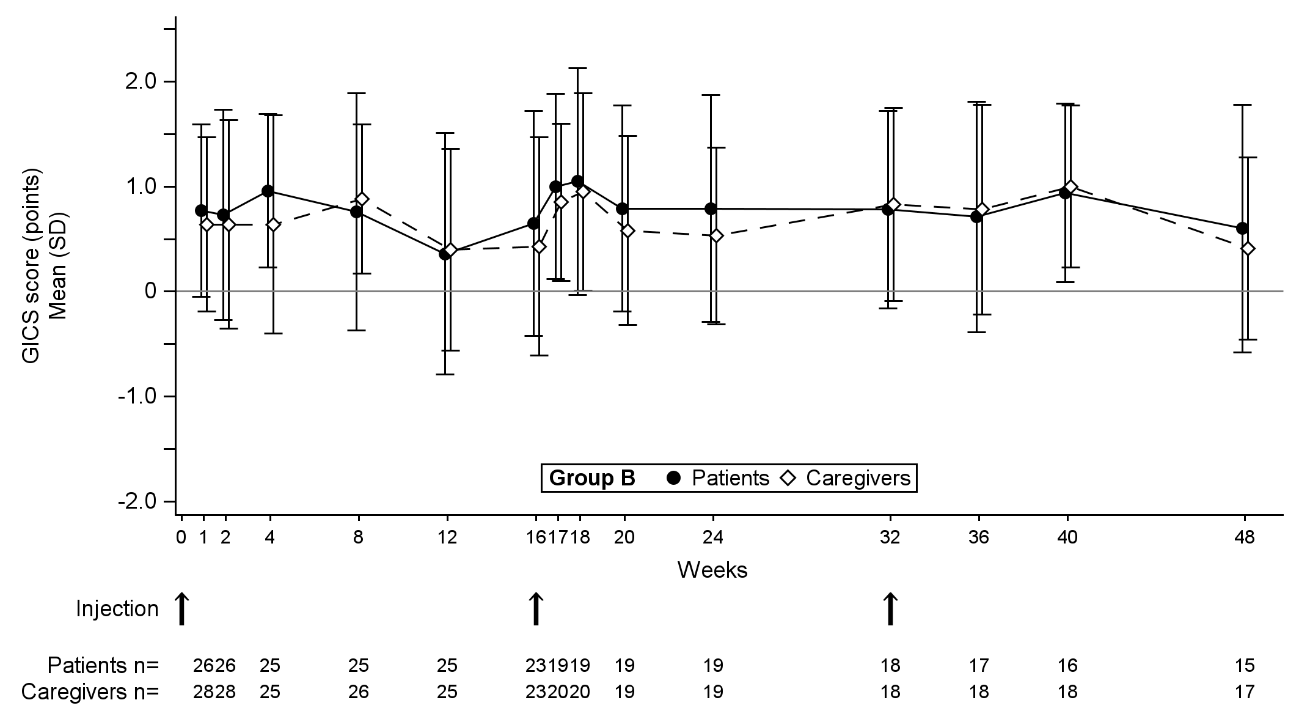


Abbreviations: GICS, Global Impression of Change Scale; SD, standard deviation.

## **Table S1.** GICS scores by patients and caregivers (full analysis set, groups A and B)

|  | Weeks | 0 | 1 | 2 | 4 | 8 | 12 | 16 | 17 | 18 | 20 | 24 | 32 | 36 | 40 | 48 |
| --- | --- | --- | --- | --- | --- | --- | --- | --- | --- | --- | --- | --- | --- | --- | --- | --- |
| Group A  Patients | Mean | 0.00 | 0.46 | 0.58 | 0.55 | 0.33 | 0.60 | 0.56 | 0.63 | 0.58 | 0.60 | 0.38 | 0.52 | 0.44 | 0.34 | 0.50 |
|  | SD | 0.00 | 0.96 | 1.05 | 1.19 | 1.22 | 0.97 | 1.06 | 0.96 | 0.99 | 0.96 | 1.00 | 0.99 | 1.07 | 1.03 | 1.03 |
| Group A  Caregiver | Mean | 0.00 | 0.30 | 0.38 | 0.32 | 0.13 | 0.43 | 0.35 | 0.84 | 0.89 | 0.90 | 0.8 | 0.86 | 0.67 | 0.60 | 0.79 |
|  | SD | 0.00 | 0.93 | 0.92 | 1.32 | 1.32 | 1.04 | 1.07 | 0.76 | 0.81 | 1.14 | 0.83 | 0.85 | 1.15 | 0.94 | 0.98 |
| Group B  Patients | Mean | 0.00 | 0.77 | 0.73 | 0.96 | 0.76 | 0.36 | 0.65 | 1.00 | 1.05 | 0.79 | 0.79 | 0.78 | 0.71 | 0.94 | 0.6 |
|  | SD | 0.00 | 0.82 | 1.00 | 0.73 | 1.13 | 1.15 | 1.07 | 0.88 | 1.08 | 0.98 | 1.08 | 0.94 | 1.10 | 0.85 | 1.18 |
| Group B  Caregiver | Mean | 0.00 | 0.64 | 0.64 | 0.64 | 0.88 | 0.40 | 0.43 | 0.85 | 0.95 | 0.58 | 0.53 | 0.83 | 0.78 | 1.00 | 0.41 |
|  | SD | 0.00 | 0.83 | 0.99 | 1.04 | 0.71 | 0.96 | 1.04 | 0.75 | 0.94 | 0.90 | 0.84 | 0.92 | 1.00 | 0.77 | 0.87 |

Abbreviations: GICS, Global Impression of Change Scale; SD, standard deviation.

## **Table S2.** EQ-5D-5L (single items, full analysis set, group A)

| Parameter | Observed Value | | | | | |
| --- | --- | --- | --- | --- | --- | --- |
| Group A | N | 1 | 2 | 3 | 4 | 5 |
| Study Visit |  | n (%) | n (%) | n (%) | n (%) | n (%) |
| Mobility |  |  |  |  |  |  |
| Before adm. test | 57 | 13 (22.8) | 26 (45.6) | 6 (10.5) | 8 (14.0) | 4 (7.0) |
| Week 4 after 1st adm. | 56 | 13 (23.2) | 18 (32.1) | 14 (25.0) | 7 (12.5) | 4 (7.1) |
| Week 8 after 1st adm. | 55 | 16 (29.1) | 16 (29.1) | 10 (18.2) | 9 (16.4) | 4 (7.3) |
| Week 12 after 1st adm. | 55 | 14 (25.5) | 15 (27.3) | 10 (18.2) | 11 (20.0) | 5 (9.1) |
| Week 16 after 1st adm. | 54 | 9 (16.7) | 23 (42.6) | 9 (16.7) | 8 (14.8) | 5 (9.3) |
| Week 4 after 2nd adm. | 48 | 10 (20.8) | 19 (39.6) | 9 (18.8) | 5 (10.4) | 5 (10.4) |
| Week 16 after 2nd adm. | 48 | 10 (20.8) | 19 (39.6) | 11 (22.9) | 3 (6.3) | 5 (10.4) |
| Week 4 after 3rd adm. | 48 | 12 (25.0) | 17 (35.4) | 9 (18.8) | 7 (14.6) | 3 (6.3) |
| Week 16 after 3rd adm. | 46 | 13 (28.3) | 16 (34.8) | 9 (19.6) | 5 (10.9) | 3 (6.5) |
| Self-Care |  |  |  |  |  |  |
| Before adm. test | 57 | 21 (36.8) | 20 (35.1) | 8 (14.0) | 5 (8.8) | 3 (5.3) |
| Week 4 after 1st adm. | 56 | 16 (28.6) | 18 (32.1) | 11 (19.6) | 9 (16.1) | 2 (3.6) |
| Week 8 after 1st adm. | 55 | 15 (27.3) | 22 (40.0) | 10 (18.2) | 7 (12.7) | 1 (1.8) |
| Week 12 after 1st adm. | 55 | 16 (29.1) | 20 (36.4) | 12 (21.8) | 6 (10.9) | 1 (1.8) |
| Week 16 after 1st adm. | 54 | 11 (20.4) | 25 (46.3) | 14 (25.9) | 2 (3.7) | 2 (3.7) |
| Week 4 after 2nd adm. | 48 | 15 (31.3) | 16 (33.3) | 10 (20.8) | 6 (12.5) | 1 (2.1) |
| Week 16 after 2nd adm. | 48 | 17 (35.4) | 16 (33.3) | 10 (20.8) | 3 (6.3) | 2 (4.2) |
| Week 4 after 3rd adm. | 48 | 15 (31.3) | 15 (31.3) | 12 (25.0) | 5 (10.4) | 1 (2.1) |
| Week 16 after 3rd adm. | 46 | 16 (34.8) | 18 (39.1) | 7 (15.2) | 4 (8.7) | 1 (2.2) |
| Usual Activities |  |  |  |  |  |  |
| Before adm. test | 57 | 11 (19.3) | 25 (43.9) | 12 (21.1) | 9 (15.8) | 0 (0.0) |
| Week 4 after 1st adm. | 56 | 13 (23.2) | 21 (37.5) | 14 (25.0) | 6 (10.7) | 2 (3.6) |
| Week 8 after 1st adm. | 55 | 12 (21.8) | 22 (40.0) | 13 (23.6) | 5 (9.1) | 3 (5.5) |
| Week 12 after 1st adm. | 55 | 17 (30.9) | 16 (29.1) | 12 (21.8) | 8 (14.5) | 2 (3.6) |
| Week 16 after 1st adm. | 54 | 11 (20.4) | 23 (42.6) | 12 (22.2) | 7 (13.0) | 1 (1.9) |
| Week 4 after 2nd adm. | 48 | 14 (29.2) | 22 (45.8) | 6 (12.5) | 5 (10.4) | 1 (2.1) |
| Week 16 after 2nd adm. | 48 | 16 (33.3) | 19 (39.6) | 6 (12.5) | 5 (10.4) | 2 (4.2) |
| Week 4 after 3rd adm. | 48 | 14 (29.2) | 17 (35.4) | 9 (18.8) | 7 (14.6) | 1 (2.1) |
| Week 16 after 3rd adm. | 46 | 13 (28.3) | 22 (47.8) | 5 (10.9) | 6 (13.0) | 0 (0.0) |
| Pain/Discomfort |  |  |  |  |  |  |
| Before adm. test | 57 | 21 (36.8) | 19 (33.3) | 14 (24.6) | 3 (5.3) | 0 (0.0) |
| Week 4 after 1st adm. | 56 | 19 (33.9) | 21 (37.5) | 11 (19.6) | 5 (8.9) | 0 (0.0) |
| Week 8 after 1st adm. | 55 | 26 (47.3) | 18 (32.7) | 11 (20.0) | 0 (0.0) | 0 (0.0) |
| Week 12 after 1st adm. | 55 | 23 (41.8) | 16 (29.1) | 13 (23.6) | 2 (3.6) | 1 (1.8) |
| Week 16 after 1st adm. | 54 | 20 (37.0) | 22 (40.7) | 9 (16.7) | 2 (3.7) | 1 (1.9) |
| Week 4 after 2nd adm. | 48 | 17 (35.4) | 20 (41.7) | 7 (14.6) | 4 (8.3) | 0 (0.0) |
| Week 16 after 2nd adm. | 48 | 14 (29.2) | 18 (37.5) | 12 (25.0) | 3 (6.3) | 1 (2.1) |
| Week 4 after 3rd adm. | 48 | 16 (33.3) | 20 (41.7) | 8 (16.7) | 4 (8.3) | 0 (0.0) |
| Week 16 after 3rd adm. | 46 | 16 (34.8) | 18 (39.1) | 10 (21.7) | 2 (4.3) | 0 (0.0) |
| Anxiety/Depression |  |  |  |  |  |  |
| Before adm. test | 57 | 26 (45.6) | 24 (42.1) | 3 (5.3) | 4 (7.0) | 0 (0.0) |
| Week 4 after 1st adm. | 56 | 36 (64.3) | 12 (21.4) | 6 (10.7) | 2 (3.6) | 0 (0.0) |
| Week 8 after 1st adm. | 55 | 36 (65.5) | 10 (18.2) | 6 (10.9) | 3 (5.5) | 0 (0.0) |
| Week 12 after 1st adm. | 55 | 32 (58.2) | 15 (27.3) | 6 (10.9) | 2 (3.6) | 0 (0.0) |
| Week 16 after 1st adm. | 54 | 33 (61.1) | 14 (25.9) | 5 (9.3) | 2 (3.7) | 0 (0.0) |
| Week 4 after 2nd adm. | 48 | 34 (70.8) | 9 (18.8) | 2 (4.2) | 3 (6.3) | 0 (0.0) |
| Week 16 after 2nd adm. | 48 | 28 (58.3) | 13 (27.1) | 6 (12.5) | 1 (2.1) | 0 (0.0) |
| Week 4 after 3rd adm. | 48 | 32 (66.7) | 11 (22.9) | 2 (4.2) | 3 (6.3) | 0 (0.0) |
| Week 16 after 3rd adm. | 46 | 32 (69.6) | 11 (23.9) | 2 (4.3) | 1 (2.2) | 0 (0.0) |

For Mobility, 1: I have no problems in walking about, 2: I have slight problems in walking about, 3: I have moderate problems in walking about, 4: I have severe problems in walking about, 5: I am unable to walk about.

For Self-Care, 1: I have no problems washing or dressing myself, 2: I have slight problems washing or dressing myself, 3: I have moderate problems washing or dressing myself, 4: I have severe problems washing or dressing myself, 5: I am unable to wash or dress myself.

For Usual Activities, 1: I have no problems doing my usual activities, 2: I have slight problems doing my usual activities, 3: I have moderate problems doing my usual activities, 4: I have severe problems doing my usual activities, 5: I am unable to do my usual activities.

For Pain/Discomfort, 1: I have no pain or discomfort, 2: I have slight pain or discomfort, 3: I have moderate pain or discomfort, 4: I have severe pain or discomfort, 5: I have extreme pain or discomfort.

For Anxiety/Depression, 1: I am not anxious or depressed, 2: I am slightly anxious or depressed, 3: I am moderately anxious or depressed, 4: I am severely anxious or depressed, 5: I am extremely anxious or depressed.

Abbreviations: adm., administration; EQ-5D-5L, EuroQol 5 dimensions 5-level.

## **Table S3.** EQ-5D-5L (Visual Analog Scale, full analysis set, group A)

| Parameter | Observed Value | | | Change from Baseline | | |
| --- | --- | --- | --- | --- | --- | --- |
| Group | n | Mean | SD | n | Mean | SD |
| Study Visit |  |  |  |  |  |  |
| VAS |  |  |  |  |  |  |
| Group A |  |  |  |  |  |  |
| Before adm. test | 57 | 63.44 | 16.62 | - | - | - |
| Week 4 after 1st adm. | 56 | 65.64 | 16.23 | 56 | 2.50 | 15.47 |
| Week 8 after 1st adm. | 55 | 65.24 | 16.70 | 55 | 1.49 | 19.23 |
| Week 12 after 1st adm. | 55 | 64.16 | 17.85 | 55 | 0.42 | 15.35 |
| Week 16 after 1st adm. | 54 | 67.33 | 15.75 | 54 | 3.70 | 16.33 |
| Week 4 after 2nd adm. | 48 | 66.08 | 18.09 | 48 | 2.63 | 18.24 |
| Week 16 after 2nd adm. | 48 | 68.15 | 15.96 | 48 | 4.69 | 18.71 |
| Week 4 after 3rd adm. | 48 | 67.13 | 18.70 | 48 | 3.67 | 20.70 |
| Week 16 after 3rd adm. | 46 | 67.20 | 17.48 | 46 | 3.04 | 17.04 |

Abbreviations: adm., administration; EQ-5D-5L, EuroQol 5 dimensions 5-level; SD, standard deviation: VAS, Visual Analog Scale.

## **Table S4.** Number (%) of patients with adverse events during treatment with incobotulinumtoxinA after each stage of administration (safety analysis set)

|  | 1st Administration | | | 2nd Administration | | | 3rd Administration | | | Overall | | |
| --- | --- | --- | --- | --- | --- | --- | --- | --- | --- | --- | --- | --- |
|  | Group A | Group B | Total | Group A | Group B | Total | Group A | Group B | Total | Group A | Group B | Total |
|  | (N = 58) | (N = 34) | (N = 92) | (N = 48) | (N = 25) | (N = 73) | (N = 48) | (N = 21) | (N = 69) | (N = 58) | (N = 34) | (N = 92) |
|  | n (%) | n (%) | n (%) | n (%) | n (%) | n (%) | n (%) | n (%) | n (%) | n (%) | n (%) | n (%) |
| Any adverse events | 25 (43.1) | 18 (52.9) | 43 (46.7) | 22 (45.8) | 12 (48.0) | 34 (46.6) | 28 (58.3) | 11 (52.4) | 39 (56.5) | 46 (79.3) | 30 (88.2) | 76 (82.6) |
| Any treatment-related adverse events | 7 (12.1) | 8 (23.5) | 15 (16.3) | 1 (2.1) | 3 (12.0) | 4 (5.5) | 2 (4.2) | 0 | 2 (2.9) | 9 (15.5) | 11 (32.4) | 20 (21.7) |
| Serious |  |  |  |  |  |  |  |  |  |  |  |  |
| Adverse events | 6 (10.3) | 5 (14.7) | 11 (12.0) | 4 (8.3) | 3 (12.0) | 7 (9.6) | 2 (4.2) | 3 (14.3) | 5 (7.2) | 11 (19.0) | 10 (29.4) | 21 (22.8) |
| Treatment-related adverse events | 2 (3.4) | 1 (2.9) | 3 (3.3) | 0 | 0 | 0 | 0 | 0 | 0 | 2 (3.4) | 1 (2.9) | 3 (3.3) |
| Leading to discontinuation |  |  |  |  |  |  |  |  |  |  |  |  |
| Adverse events | 2 (3.4) | 5 (14.7) | 7 (7.6) | 0 | 2 (8.0) | 2 (2.7) | 0 | 0 | 0 | 2 (3.4) | 7 (20.6) | 9 (9.8) |
| Treatment-related adverse events | 2 (3.4) | 4 (11.8) | 6 (6.5) | 0 | 1 (4.0) | 1 (1.4) | 0 | 0 | 0 | 2 (3.4) | 5 (14.7) | 7 (7.6) |
| Leading to death |  |  |  |  |  |  |  |  |  |  |  |  |
| Adverse events | 0 | 1 (2.9) | 1 (1.1) | 0 | 1 (4.0) | 1 (1.4) | 1 (2.1) | 0 | 1 (1.4) | 1 (1.7) | 2 (5.9) | 3 (3.3) |
| Treatment-related adverse events | 0 | 0 | 0 | 0 | 0 | 0 | 0 | 0 | 0 | 0 | 0 | 0 |

MedDRA, Version 26.0, was used for coding adverse events. Treatment-related adverse events are adverse events determined to be related to the administration of incobotulinumtoxinA.

Abbreviations: MedDRA, Medical Dictionary for Regulatory Activities.

## **Table S5.** Treatment-related adverse events in System Organ Class and Preferred Term of 2% or more (safety analysis set)

| System Organ Class | 1st Administration | | | 2nd Administration | | | 3rd Administration | | | Overall | | |
| --- | --- | --- | --- | --- | --- | --- | --- | --- | --- | --- | --- | --- |
| Preferred Term | Group A | Group B | Total | Group A | Group B | Total | Group A | Group B | Total | Group A | Group B | Total |
|  | (N = 58) | (N = 34) | (N = 92) | (N = 48) | (N = 25) | (N = 73) | (N = 48) | (N = 21) | (N = 69) | (N = 58) | (N = 34) | (N = 92) |
|  | n (%) | n (%) | n (%) | n (%) | n (%) | n (%) | n (%) | n (%) | n (%) | n (%) | n (%) | n (%) |
| Gastrointestinal disorders | 6 (10.3) | 5 (14.7) | 11 (12.0) | 0 (0.0) | 3 (12.0) | 3 (4.1) | 1 (2.1) | 0 (0.0) | 1 (1.4) | 6 (10.3) | 8 (23.5) | 14 (15.2) |
| Dry mouth | 4 (6.9) | 2 (5.9) | 6 (6.5) | 0 (0.0) | 2 (8.0) | 2 (2.7) | 0 (0.0) | 0 (0.0) | 0 (0.0) | 4(6.9) | 4 (11.8) | 8 (8.7) |
| Dysphagia | 4 (6.9) | 3 (8.8) | 7 (7.6) | 0 (0.0) | 1 (4.0) | 1 (1.4) | 0 (0.0) | 0 (0.0) | 0 (0.0) | 4 (6.9) | 4 (11.8) | 8 (8.7) |
| Musculoskeletal and connective tissue disorders | 2 (3.4) | 0 (0.0) | 2 (2.2) | 1 (2.1) | 0 (0.0) | 1 (1.4) | 0 (0.0) | 0 (0.0) | 0 (0.0) | 3 (5.2) | 0 (0.0) | 3 (3.3) |
| Mastication disorder | 1 (1.7) | 0 (0.0) | 1 (1.1) | 0 (0.0) | 0 (0.0) | 0 (0.0) | 0 (0.0) | 0 (0.0) | 0 (0.0) | 1 (1.7) | 0 (0.0) | 1 (1.1) |
| Muscle atrophy | 0 (0.0) | 0 (0.0) | 0 (0.0) | 1 (2.1) | 0 (0.0) | 1 (1.4) | 0 (0.0) | 0 (0.0) | 0 (0.0) | 1 (1.7) | 0 (0.0) | 1 (1.1) |
| Immobilisation syndrome | 1 (1.7) | 0 (0.0) | 1 (1.1) | 0 (0.0) | 0 (0.0) | 0 (0.0) | 0 (0.0) | 0 (0.0) | 0 (0.0) | 1 (1.7) | 0 (0.0) | 1 (1.1) |
| General disorders and administration site conditions | 0 (0.0) | 2 (5.9) | 2 (2.2) | 0 (0.0) | 0 (0.0) | 0 (0.0) | 1 (2.1) | 0 (0.0) | 1 (1.4) | 1 (1.7) | 2 (5.9) | 3 (3.3) |
| Thirst | 0 (0.0) | 2 (5.9) | 2 (2.2) | 0 (0.0) | 0 (0.0) | 0 (0.0) | 1 (2.1) | 0 (0.0) | 1 (1.4) | 1 (1.7) | 2 (5.9) | 3 (3.3) |

Patients with one or more adverse events within a level of MedDRA term are counted only once in that level.

Percentages are based on the number of patients in the safety analysis set for each group.

MedDRA, Version 26.0, was used for coding adverse events.

Abbreviations: MedDRA, Medical Dictionary for Regulatory Activities.

## **Table S6.** Adverse events leading to discontinuation of incobotulinumtoxinA (safety analysis set)

| System Organ Class | 1st Administration | | | 2nd Administration | | | 3rd Administration | | | Overall | | |
| --- | --- | --- | --- | --- | --- | --- | --- | --- | --- | --- | --- | --- |
| Preferred Term | Group A | Group B | Total | Group A | Group B | Total | Group A | Group B | Total | Group A | Group B | Total |
|  | (N = 58) | (N = 34) | (N = 92) | (N = 48) | (N = 25) | (N = 73) | (N = 48) | (N = 21) | (N = 69) | (N = 58) | (N = 34) | (N = 92) |
|  | n (%) | n (%) | n (%) | n (%) | n (%) | n (%) | n (%) | n (%) | n (%) | n (%) | n (%) | n (%) |
| Total | 2 (3.4) | 5 (14.7) | 7 (7.6) | 0 (0.0) | 2 (8.0) | 2 (2.7) | 0 (0.0) | 0 (0.0) | 0 (0.0) | 2 (3.4) | 7 (20.6) | 9 (9.8) |
| Infections and infestations | 0 (0.0) | 1 (2.9) | 1 (1.1) | 0 (0.0) | 1 (4.0) | 1 (1.4) | 0 (0.0) | 0 (0.0) | 0 (0.0) | 0 (0.0) | 2 (5.9) | 2 (2.2) |
| Pneumonia aspiration | 0 (0.0) | 1 (2.9) | 1 (1.1) | 0 (0.0) | 1 (4.0) | 1 (1.4) | 0 (0.0) | 0 (0.0) | 0 (0.0) | 0 (0.0) | 2 (5.9) | 2 (2.2) |
| Blood and lymphatic system disorders | 0 (0.0) | 1 (2.9) | 1 (1.1) | 0 (0.0) | 0 (0.0) | 0 (0.0) | 0 (0.0) | 0 (0.0) | 0 (0.0) | 0 (0.0) | 1 (2.9) | 1 (1.1) |
| Thrombocytopenia | 0 (0.0) | 1 (2.9) | 1 (1.1) | 0 (0.0) | 0 (0.0) | 0 (0.0) | 0 (0.0) | 0 (0.0) | 0 (0.0) | 0 (0.0) | 1 (2.9) | 1 (1.1) |
| Nervous system disorders | 0 (0.0) | 1 (2.9) | 1 (1.1) | 0 (0.0) | 0 (0.0) | 0 (0.0) | 0 (0.0) | 0 (0.0) | 0 (0.0) | 0 (0.0) | 1 (2.9) | 1 (1.1) |
| Parkinson's disease | 0 (0.0) | 1 (2.9) | 1 (1.1) | 0 (0.0) | 0 (0.0) | 0 (0.0) | 0 (0.0) | 0 (0.0) | 0 (0.0) | 0 (0.0) | 1 (2.9) | 1 (1.1) |
| Gastrointestinal disorders | 2 (3.4) | 4 (11.8) | 6 (6.5) | 0 (0.0) | 1 (4.0) | 1 (1.4) | 0 (0.0) | 0 (0.0) | 0 (0.0) | 2 (3.4) | 5 (14.7) | 7 (7.6) |
| Dry mouth | 0 (0.0) | 1 (2.9) | 1 (1.1) | 0 (0.0) | 0 (0.0) | 0 (0.0) | 0 (0.0) | 0 (0.0) | 0 (0.0) | 0 (0.0) | 1 (2.9) | 1 (1.1) |
| Dysphagia | 2 (3.4) | 3 (8.8) | 5 (5.4) | 0 (0.0) | 1 (4.0) | 1 (1.4) | 0 (0.0) | 0 (0.0) | 0 (0.0) | 2 (3.4) | 4 (11.8) | 6 (6.5) |

Patients with one or more adverse events within a level of MedDRA term are counted only once in that level.

Percentages are based on the number of patients in the safety analysis set for each group.

MedDRA, Version 26.0, was used for coding adverse events.

Abbreviations: MedDRA, Medical Dictionary for Regulatory Activities.

## **Table S7.** Treatment-related adverse events leading to discontinuation of incobotulinumtoxinA (safety analysis set)

| System Organ Class | 1st Administration | | | 2nd Administration | | | 3rd Administration | | | Overall | | |
| --- | --- | --- | --- | --- | --- | --- | --- | --- | --- | --- | --- | --- |
| Preferred Term | Group A | Group B | Total | Group A | Group B | Total | Group A | Group B | Total | Group A | Group B | Total |
|  | (N = 58) | (N = 34) | (N = 92) | (N = 48) | (N = 25) | (N = 73) | (N = 48) | (N = 21) | (N = 69) | (N = 58) | (N = 34) | (N = 92) |
|  | n (%) | n (%) | n (%) | n (%) | n (%) | n (%) | n (%) | n (%) | n (%) | n (%) | n (%) | n (%) |
| Total | 2 (3.4) | 4 (11.8) | 6 (6.5) | 0 (0.0) | 1 (4.0) | 1 (1.4) | 0 (0.0) | 0 (0.0) | 0 (0.0) | 2 (3.4) | 5 (14.7) | 7 (7.6) |
| Infections and infestations | 0 (0.0) | 1 (2.9) | 1 (1.1) | 0 (0.0) | 0 (0.0) | 0 (0.0) | 0 (0.0) | 0 (0.0) | 0 (0.0) | 0 (0.0) | 1 (2.9) | 1 (1.1) |
| Pneumonia aspiration | 0 (0.0) | 1 (2.9) | 1 (1.1) | 0 (0.0) | 0 (0.0) | 0 (0.0) | 0 (0.0) | 0 (0.0) | 0 (0.0) | 0 (0.0) | 1 (2.9) | 1 (1.1) |
| Gastrointestinal disorders | 2 (3.4) | 4 (11.8) | 6 (6.5) | 0 (0.0) | 1 (4.0) | 1 (1.4) | 0 (0.0) | 0 (0.0) | 0 (0.0) | 2 (3.4) | 5 (14.7) | 7 (7.6) |
| Dry mouth | 0 (0.0) | 1 (2.9) | 1 (1.1) | 0 (0.0) | 0 (0.0) | 0 (0.0) | 0 (0.0) | 0 (0.0) | 0 (0.0) | 0 (0.0) | 1 (2.9) | 1 (1.1) |
| Dysphagia | 2 (3.4) | 3 (8.8) | 5 (5.4) | 0 (0.0) | 1 (4.0) | 1 (1.4) | 0 (0.0) | 0 (0.0) | 0 (0.0) | 2 (3.4) | 4 (11.8) | 6 (6.5) |

Patients with one or more adverse events within a level of MedDRA term are counted only once in that level.

Percentages are based on the number of patients in the safety analysis set for each group.

MedDRA, Version 26.0, was used for coding adverse events.

Abbreviations: MedDRA, Medical Dictionary for Regulatory Activities.

## **Table S8.** Serious treatment-related adverse events (safety analysis set)

| System Organ Class | 1st Administration | | | 2nd Administration | | | 3rd Administration | | | Overall | | |
| --- | --- | --- | --- | --- | --- | --- | --- | --- | --- | --- | --- | --- |
| Preferred Term | Group A | Group B | Total | Group A | Group B | Total | Group A | Group B | Total | Group A | Group B | Total |
|  | (N = 58) | (N = 34) | (N = 92) | (N = 48) | (N = 25) | (N = 73) | (N = 48) | (N = 21) | (N = 69) | (N = 58) | (N = 34) | (N = 92) |
|  | n (%) | n (%) | n (%) | n (%) | n (%) | n (%) | n (%) | n (%) | n (%) | n (%) | n (%) | n (%) |
| Total | 2 (3.4) | 1 (2.9) | 3 (3.3) | 0 (0.0) | 0 (0.0) | 0 (0.0) | 0 (0.0) | 0 (0.0) | 0 (0.0) | 2 (3.4) | 1 (2.9) | 3 (3.3) |
| Infections and infestations | 0 (0.0) | 1 (2.9) | 1 (1.1) | 0 (0.0) | 0 (0.0) | 0 (0.0) | 0 (0.0) | 0 (0.0) | 0 (0.0) | 0 (0.0) | 1 (2.9) | 1 (1.1) |
| Pneumonia aspiration | 0 (0.0) | 1 (2.9) | 1 (1.1) | 0 (0.0) | 0 (0.0) | 0 (0.0) | 0 (0.0) | 0 (0.0) | 0 (0.0) | 0 (0.0) | 1 (2.9) | 1 (1.1) |
| Gastrointestinal disorders | 2 (3.4) | 0 (0.0) | 2 (2.2) | 0 (0.0) | 0 (0.0) | 0 (0.0) | 0 (0.0) | 0 (0.0) | 0 (0.0) | 2 (3.4) | 0 (0.0) | 2 (2.2) |
| Dysphagia | 2 (3.4) | 0 (0.0) | 2 (2.2) | 0 (0.0) | 0 (0.0) | 0 (0.0) | 0 (0.0) | 0 (0.0) | 0 (0.0) | 2 (3.4) | 0 (0.0) | 2 (2.2) |

Patients with one or more adverse events within a level of MedDRA term are counted only once in that level.

Percentages are based on the number of patients in the safety analysis set for each group.

MedDRA, Version 26.0, was used for coding adverse events.

Abbreviations: MedDRA, Medical Dictionary for Regulatory Activities.
