# Supplementary material for: Efficacy and Safety of IncobotulinumtoxinA for Treatment of Sialorrhea: A Multicenter, Phase 3 Study in Japan
Source: Mov Disord Clin Pract. 2025 Aug 7;13(1):120–30. doi: 10.1002/mdc3.70259 (PMC12839494; doi:10.1002/mdc3.70259)
Supplement: Supplementary file 2 — Data S1: Graphical abstract (Japanese) [file MDC3-13-120-s001.pdf]

日本人を対象とした第3相単群臨床試験

慢性流涎（唾液過多）の患者さん**92**名が参加しました

以下の患者さんが試験に参加しました

- パーキンソン病、多系統萎縮症、進行性核上麻痺、大脳皮質基底核変性症、脳卒中、外傷性脳損傷、脳性麻痺、筋萎縮性側索硬化症、筋ジストロフィーなどの病気により、慢性流涎（唾液過多）になった患者さん

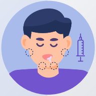

注射した部位

インコボツリヌストキシンA（100単位）

- 16週ごとに48週間にわたっての投与
- 左右の耳の下とあごの下の唾液腺の合計4カ所に注射しました

結果

有効性

- 唾液の出る量が48週間にわたって抑えられました
- 唾液が増えてしまうことによる症状が、48週間にわたって改善しました

安全性

- もっとも多く認められた副作用は、口内の乾燥と嚥下障害(飲み込みにくくなること)でした
- 新たな安全性の問題は認められませんでした

パーキンソン病などの神経疾患により慢性流涎（唾液過多）になった日本人患者さんに、インコボツリヌストキシンAを注射した結果、有効性と安全性が示されました。48週間にわたって唾液の出る量が抑えられ、唾液が増えることによる症状も改善しました。副作用は、患者さんにとって許容できるもので、安全性に関して新しい問題はありませんでした。
